# Editorial: Nutrition and wellbeing: how do energy intake, fasting and prudent diets affect mental health

**DOI:** 10.3389/fnut.2024.1461415

**Published:** 2024-07-31

**Authors:** Achraf Ammar, Khaled Trabelsi, Omar Hammouda, Cain C. T. Clark

**Affiliations:** ^1^Department of Training and Movement Science, Institute of Sport Science, Johannes Gutenberg-University Mainz, Mainz, Germany; ^2^Research Laboratory, Molecular Bases of Human Pathology, LR19ES13, Faculty of Medicine, University of Sfax, Sfax, Tunisia; ^3^High Institute of Sport and Physical Education of Sfax, University of Sfax, Sfax, Tunisia; ^4^Interdisciplinary Laboratory in Neurosciences, Physiology and Psychology: Physical Activity, Health and Learning (LINP2), UFR STAPS (Faculty of Sport Sciences), Paris Nanterre University, Nanterre, France; ^5^Research Laboratory: Education, Motricity, Sport and Health (EM2S), LR15JS01, High Institute of Sport and Physical Education, University of Sfax, Sfax, Tunisia; ^6^College of Life Sciences, Birmingham City University, Birmingham, United Kingdom

**Keywords:** food, caloric intake, healthy lifestyle, mental health, psychology, nutritional psychology, psycho-nutrition, health information technology

The field of psycho-nutrition has garnered considerable interest with emerging evidence espousing the protective role of healthy diets, reduced energy consumption, and different fasting regimens on mental health (1). Calorie restriction (CR) and various fasting regimens have been identified as some of the most effective non-pharmacological interventions to enhance cardiovascular and metabolic health. However, their effects on mental health remain controversial due to variations in intensity, duration, and period of CR/fasting. While many studies have demonstrated that CR can improve quality of life and reduce risk factors for psychiatric diseases, such as depression, excessive CR may impair cognitive abilities and overall quality of life, leading to negative mood states (2, 3). Similarly, reducing dietary fat may adversely affect mood, whereas adopting prudent diets, like the Mediterranean, MIND, and DASH, which emphasize healthy fats and carbohydrates, can reduce depressive symptoms and enhance overall wellbeing (4, 5). Fasting interventions have also gained popularity, though their effects on mental health are inconsistent, with some studies reporting psychological improvements and others noting worsened mood and increased depression (6).

The psychological safety of these dietary regimens may limit their application in both general and athletic populations. Indeed, it remains unclear whether fasting and/or CR interventions decrease or increase depressive symptoms. Additionally, the relationship between CR, various fasting regimens, the gut-brain axis, and mental health is not well understood. Another significant question is the effectiveness of combined dietary interventions (e.g., fasting and CR) in improving depressive symptoms and mental wellbeing. Moreover, the interplay between diet, exercise, sleep, and stress, especially in athletic populations, requires further exploration. Epidemiological data highlight the association between diet and mental health across different body systems, but provide limited insights into causality, bidirectional interactions, and underlying mechanisms (7, 8).

This Research Topic aims to provide a comprehensive view of studies related to the bidirectional interactions between nutrition and mental health. It focuses on review- and interventional-based studies investigating the effects of CR, various fasting regimens, and prudent diets on mental health, behavior, and sleep quality in the general population, athletes, and patients. Additionally, it considers the impact of various psychiatric conditions on CR and diet. A better understanding of these psychological effects will help optimize the practice of these dietary regimens and maximize their health benefits. In total, eight papers have been published in the present Research Topic, “*Nutrition and wellbeing: how do energy intake, fasting, and prudent diets affect mental health*,” which collectively cover a broad scope of research in this emerging field.

## Summary of main findings

The eight studies summarized herein provide valuable and novel insights into how various dietary regimens, including CR, fasting, and specific nutrient intakes, influence mental health outcomes across different populations.

Zhang L. et al. conducted a randomized controlled trial (RCT) on a calorie-restricted diet in obese women with schizophrenia, and revealed significant improvements in weight-related measures, but limited metabolic benefits, highlighting the complex interplay between physical and mental health in clinical populations. Bougrine et al. investigated the timing of Suhoor during Ramadan fasting among female athletes and demonstrated that late Suhoor intake preserved cognitive performance better than early Suhoor, suggesting the importance of nutrient timing in optimizing athletic performance and cognitive functions during fasting.

Survey-based studies by Ra and Zhang L. et al. further illustrated the significant associations between diet and mental health. Unhealthy diets, such as the combined consumption of sugar-sweetened beverages (SSBs) and fast foods (Ra's study) and diets with inflammatory potential (Zhang Y. et al.), were linked to heightened mental strain. Similarly, Shen et al. demonstrated an inverse relationship between calcium consumption and depressive symptoms among American adults. These findings emphasize the need for targeted interventions to reduce the consumption of SSBs and fast foods in school and community settings, highlight the potential of anti-inflammatory diets to mitigate depression and reduce mortality risk, particularly in non-cancer populations, and advocate for greater awareness and adequate intake of dietary calcium to support mental health, respectively.

Review-based studies also contribute significant insights. Hosseini et al. critically reviewed the effects of fasting diets (FDs) on eating behaviors, sleep, mood, and overall wellbeing, highlighting the potential mental health benefits of fasting diets while calling for more research on their impacts on the gut-brain axis. In a mini review on the low fermentable oligosaccharide, disaccharide, monosaccharide, and polyol (FODMAP) diet, O'Neill et al. reported positive effects on mental health through gut microbiota modulation, suggesting promising avenues for treating major depressive disorder (MDD) with dietary interventions. Bernhardt et al. (9) conducted a systematic review on probiotics and underscored their role in alleviating alcohol addiction and alcoholic liver disease (ALD) by regulating neurotransmitter pathways and reducing neuroinflammation, proposing probiotics as a viable therapeutic strategy for mental health improvement in these conditions.

## Review-based studies

Hosseini et al. conducted a comprehensive critical review exploring the effects of FDs on eating behaviors, sleep, mood, and overall wellbeing. This review highlighted that, while fasting diets have gained significant attention for their potential health benefits, there remains a lack of clarity regarding which fasting regimen provides the most advantageous effects. The review revealed that fasting can positively impact mental health by potentially enhancing mood and modifying eating behaviors, yet the consistency and comparability of trials are limited. Furthermore, the authors emphasized the need for more research to understand the impacts of various fasting regimes on the gut-brain axis, which could provide new treatment avenues for resistant anxiety and depression. The review also suggested that fasting diets could have lasting effects on physiological parameters, thereby offering a novel approach to the prevention and treatment of issues related to eating behaviors and associated comorbidities.

In a mini-review, O'Neill et al. explored how a FODMAP elimination diet affects mental health through the gut-brain axis. Their review highlighted that changes induced by a low FODMAP diet in gut microbiota composition were associated with improved mental health outcomes, including reduced anxiety and depression symptoms. The review underscored the importance of the gut microbiome in mediating the effects of a low FODMAP diet on the brain, suggesting that alterations in gut bacteria due to dietary intervention could lead to significant mental health benefits. Specific mechanisms discussed include the modulation of gut-derived neurotransmitters, reduction in systemic inflammation, and improved gut barrier function, all of which contribute to better mental health. The review provided preliminary evidence that a low FODMAP diet might drive beneficial changes in microbiota that also positively impact individuals with MDD, warranting further research to assess whether this diet can effectively treat MDD through targeted microbiota modification.

The systematic review by Bernhardt et al. (9) assessed the role of probiotics in alleviating alcohol addiction and ALD. Their review found that probiotics helped regulate neurotransmitter pathways, reduce neuroinflammation, and restore gut flora balance. Probiotic treatments successfully corrected microbiota imbalances, decreased intestinal permeability, and prevented the translocation of bacterial components, such as lipopolysaccharides (LPS), into the bloodstream. These treatments were shown to decrease systemic inflammation, improve gut permeability, and modulate key neurotransmitters associated with addiction behaviors, particularly those connected to GABA, glutamate, and dopamine. Furthermore, probiotics altered the expression of neurotransmitter signaling and dopamine receptors, highlighting their potential in treating addiction. These findings support the potential of probiotics as a therapeutic strategy for alcohol addiction and associated neuroinflammatory conditions. The review also highlighted specific strains of probiotics that were effective in these contexts, suggesting targeted probiotic therapy as a promising intervention for improving mental health in individuals with ALD and those undergoing alcohol deaddiction.

## Interventional studies

Zhang L. et al. conducted a RCT to evaluate the effects of a calorie-restricted diet (CRD) on weight loss and metabolic health in hospitalized obese women with schizophrenia over a 4-week period. The findings demonstrated significant improvements in weight-related measures with reductions in body weight, body mass index, waist circumference, and fat mass in the intervention group compared to the control group. However, compared to the control group, the experimental group did not experience significantly improved metabolic parameters such as triglycerides and cholesterol levels. These findings suggest that while CRD can be effective for weight loss, its metabolic benefits may be limited. The study involved close monitoring of dietary intake and adherence, highlighting the challenges and feasibility of implementing CRD in clinical populations with complex psychiatric and metabolic needs.

In a different population, focusing on healthy female athletes, Bougrine et al. conducted an RCT aiming to evaluate the link between the timing of the last meal “Suhoor” and diurnal variations in cognitive performance during Ramadan fasting (RIF). The athletes were tested at three different times of day across four periods: the 10 days preceding Ramadan, the final 12 days of Ramadan under early Suhoor (SEarly) and late Suhoor (SLate) conditions, and the 10 days immediately after Ramadan. Cognitive performance was assessed along with sleep quality and daily dietary intake. The results indicated that sleep quality significantly declined during the Ramadan and post-Ramadan periods, though total dietary intake and oral temperature remained unchanged. Compared to the pre-Ramadan period, cognitive performance in the afternoon declined under both SEarly and SLate conditions, with midday performance also decreasing during SLate. Morning performance was unaffected in both conditions. Notably, SLate resulted in better cognitive performance at midday and the afternoon compared to SEarly. The study concludes that the timing of nutrient intake significantly affects diurnal fluctuations in cognitive functions during Ramadan fasting, particularly around noon and in the afternoon. The findings suggest that consuming Suhoor later in the night helps preserve optimal morning cognitive abilities and prevents cognitive impairment during the fasted state at midday and in the afternoon, thus potentially enhancing overall athletic performance. The study highlights the need for future research to explore the effects of RIF on diverse demographic groups, considering additional factors such as training load, hydration status, and psychological stress levels to provide a more comprehensive understanding of optimal nutrient timing for cognitive performance.

## Survey-based studies

Ra conducted a survey-based study to evaluate the combined effects of SSBs and fast-food consumption on the mental health of Korean high school students. Utilizing secondary data from the 17th Korea Youth Risk Behavior Web-based Survey, which included 24,006 participants, the study employed complex sampling analysis for statistical evaluation. The findings indicated that the combined consumption of more than medium amounts of SSBs and fast foods was significantly associated with increased stress, depressive symptoms, and suicidal ideation compared to their independent consumption. Furthermore, high combined consumption of SSBs and varying levels of fast foods showed dose-dependent negative effects on these mental health parameters. The study suggested that healthcare providers in schools and communities should develop targeted interventions, such as school and community-based feeding programs and policies, to restrict SSB and fast-food consumption, thereby improving adolescents' mental health.

In another survey-based study, Zhang Y. et al. examined the association between the energy-adjusted Dietary Inflammatory Index (E-DII) score and the risks of depression and mortality, with a particular focus on cancer survivors, using data from the 2007–2018 National Health and Nutrition Examination Survey (NHANES). The study included 27,447 participants, comprising 24,694 individuals without cancer and 2,753 cancer survivors. The E-DII score, derived from a 24-h dietary recall, was used to measure dietary inflammation, while depressive symptoms were assessed using the Patient Health Questionnaire-9 (PHQ-9). Logistic regression analyses revealed that higher E-DII scores were positively associated with an increased risk of depression in both groups. Additionally, the highest quartile of E-DII scores was linked to a higher risk of all-cause and cardiovascular disease (CVD) mortality in subjects without cancer, but not in cancer survivors. Participants with depressive symptoms also exhibited higher all-cause mortality. The findings underscore the potential of anti-inflammatory diets to mitigate depression and reduce mortality risk, highlighting their clinical and public health significance. However, the study's cross-sectional design and reliance on self-reported data suggest the need for further research to confirm these associations.

Similarly, aiming to evaluate the association between dietary calcium (Ca) and depression among American adults, Shen et al. conducted a study using data from the National Health and Nutrition Examination Survey (NHANES) from 2007 to 2016. This study included 14,971 participants aged over 18 years. Dietary Ca intake was measured using a 24-h dietary recall method, and depressive symptoms were assessed using the Patient Health Questionnaire-9 (PHQ-9), with scores of ≥10 indicating depressive symptoms. The association between dietary Ca and depressive symptoms was examined using multivariate logistic regression, sensitivity analysis, and restricted cubic spline regression. The results indicated that 7.6% of the participants had depressive symptoms. After adjusting for various potential confounders, including demographic, lifestyle, and health-related factors, the study found that higher Ca intake was associated with a lower risk of depressive symptoms. Specifically, the adjusted odds ratios (ORs) for depression were lower in higher quartiles of Ca intake compared to the lowest quartile, demonstrating a significant inverse relationship. The association was linear, and no significant interactions were found except among different races. The study concluded that dietary Ca intake may be negatively associated with the risk of depressive symptoms in US adults, suggesting that increasing Ca intake may help reduce the prevalence of depressive symptoms. These findings highlight the importance of adequate Ca consumption and call for increased awareness among American adults regarding their dietary Ca intake. However, the authors note the limitations of the cross-sectional design and reliance on self-reported data, recommending further prospective studies to confirm the veracity of observed associations.

## Concluding remarks

The studies published in this Research Topic highlight the complex interactions between nutrition and mental health. They provide preliminary support for the beneficial effects of calorie restriction, various fasting regimens, and prudent diets on mental wellbeing. However, the findings also emphasize the need for caution, as the effects are nuanced and influenced by various factors, such as timing, nutrient composition, and individual health status. The present editorial highlights the necessity for future research to further elucidate the bidirectional interactions between diet and mental health, particularly through longitudinal and prospective studies that can better establish causality. Future research should also aim to elucidate the mechanisms underlying these effects, explore the role of the gut-brain axis, and determine the long-term implications of these dietary interventions. Additionally, studies should consider diverse populations, including different age groups, genders, and levels of athletic expertise, as well as to control for additional variables such as such as training load, hydration status, and psychological stress levels for study among the athletic population to provide a more comprehensive understanding of optimal dietary strategies for mental wellbeing. Overall, the research presented affirms the significant impact of nutrition on mental health and underscores the potential of dietary interventions as non-pharmacological strategies to enhance psychological wellbeing. However, the variability in study designs and outcomes calls for more rigorous and standardized approaches to validate these findings and translate them into practical recommendations for improving mental health through diet. By advancing our understanding of the bidirectional relationship between nutrition and mental health, we can develop more effective dietary strategies to promote mental wellbeing across various populations.

## Author contributions

AA: Conceptualization, Data curation, Formal analysis, Funding acquisition, Investigation, Methodology, Project administration, Resources, Software, Supervision, Validation, Visualization, Writing – original draft, Writing – review & editing. KT: Conceptualization, Data curation, Formal analysis, Funding acquisition, Investigation, Methodology, Project administration, Resources, Software, Supervision, Validation, Visualization, Writing – review & editing. OH: Validation, Visualization, Writing – review & editing. CC: Validation, Visualization, Writing – review & editing.
